# Effects of perfluorooctanoic acid exposure and heat stress on performance and liver health biomarkers in post-pubertal gilts

**DOI:** 10.1093/jas/skaf348

**Published:** 2025-10-10

**Authors:** Bridget M Buol, Collins Antwi-Boasiako, Edith J Mayorga, María Estefanía González-Alvarez, Lance H Baumgard, Aileen F Keating

**Affiliations:** Department of Animal Science, Iowa State University, Ames, IA 50014; Department of Animal Science, Iowa State University, Ames, IA 50014; Department of Animal Science, Iowa State University, Ames, IA 50014; Department of Animal Science, Iowa State University, Ames, IA 50014; Department of Animal Science, Iowa State University, Ames, IA 50014; Department of Animal Science, Iowa State University, Ames, IA 50014

**Keywords:** forever chemicals, per- and polyfluoroalkyl substances, pig

## Abstract

Objectives were to evaluate how dietary perfluorooctanoic acid (PFOA) and heat stress (HS) affect growth performance and liver health biomarkers in pigs. Crossbred post-pubertal gilts (*n* = 48; 164.0 ± 11.6 kg body weight [BW]) were randomly assigned to 1 of 4 treatments in a 2 × 2 factorial design: 1) thermoneutral (TN) control (*n* = 12; TN-CON), 2) TN and PFOA (*n* = 12; TN-PFOA), 3) HS control (*n* = 12; HS-CON), or 4) HS and PFOA (*n* = 12; HS-PFOA), and enrolled in 3 experimental periods (P). During P1 (3 d), pigs were housed in TN conditions (20.3 ± 0.1 °C) and baseline data were collected. During P2 (15 d), HS-CON and HS-PFOA pigs were exposed to cyclical HS (29.3 ± 0.1 to 31.9 ± 0.8 °C), while TN-CON and TN-PFOA remained in TN conditions. Altrenogest was administered once daily (0800 h) during P2 to synchronize estrus. In P3 (4 d), Altrenogest was withdrawn to induce estrus, while experimental treatments remained the same. PFOA (70 ng/kg BW) was orally administered once daily (0800 h) during P2 and P3, and all pigs were euthanized at the end of P3. HS increased rectal temperature, skin temperature, and respiration rate compared to TN counterparts during P2 (0.34 °C, 5.67 °C, and 22 bpm, respectively; *P* < 0.01) and P3 (0.26 °C, 6.96 °C, and 22 bpm, respectively; *P* < 0.01), and these were only marginally influenced by PFOA. During P2 and P3, HS markedly decreased feed intake (FI) and average daily gain (ADG), and PFOA tended to decrease FI and ADG; an effect most pronounced in TN conditions (410 g/d; *P* ≤ 0.08 and 320 g/d; *P* ≤ 0.09, respectively) during P2 and P3. HS decreased circulating alkaline phosphatase and alanine aminotransferase in P2 and P3 (*P* ≤ 0.01), and increased gamma-glutamyl transferase in P2 (*P* < 0.01), but these enzymes were unaffected by PFOA. There was little to no dietary treatment or environmental effects on other liver health biomarkers. HS decreased absolute and relative liver, lung, and kidney weights, and PFOA decreased absolute lung weight (11%; *P* < 0.05) and relative lung weight (0.06%; *P* = 0.09). In summary, PFOA and HS independently compromised appetite and growth, but the effects of PFOA and HS do not appear to be additive.

## Teaser

Heat stress and perfluorooctanoic acid exposure negatively affect pig performance, but do not appear to be additive. This highlights the need to identify mechanisms through which environmental stressors compromise animal health and productivity.

## Introduction

Perfluorooctanoic acid (PFOA) is one of a large group of chemicals known as per- and polyfluoroalkyl substances (PFAS). PFAS compounds have strong carbon-fluorine bonds that confer amphiphilic properties and exceptional thermal stability (Death et al., 2021). Due to their diverse industrial and consumer applications, PFAS exposures are widespread through drinking water, food products, packaging materials, cookware, textiles, detergents, indoor dust, and ambient air (Nadal and Domingo, 2014; Sjogren et al., 2016; Mastrantonio et al., 2018). Consequently, PFAS have been detected in the blood of 98% of the U.S. population (Calafat et al., 2007) and are implicated in adverse health outcomes, including hepatotoxicity, carcinogenicity, endocrine disruption, and immunotoxicity (White et al., 2011; Jha et al., 2021). Despite regulatory phase-out efforts targeting legacy PFAS, their environmental persistence and resistance to degradation have led to bioaccumulation in soil, water, and air, and this biomagnification is why they are classified as persistent organic pollutants.

Heat stress (HS) is a physiological and economic burden to both animal agriculture and human welfare. Vulnerable human populations, including infants, the elderly, and individuals engaged in prolonged physical labor, are particularly susceptible to heat-induced illnesses or heat stroke (Leon and Helwig, 2010). In livestock, HS triggers physiological adaptations that reduce profitability, cause infertility, and increase mortality and morbidity (Baumgard and Rhoads, 2013; Dickson et al., 2018; Mayorga et al., 2019). Economically, HS inflicts substantial losses in the U.S. livestock industry, with annual estimates exceeding $1 billion in the dairy sector and $300 million in beef and swine, mainly due to reduced productivity and increased health-related expenses (Baumgard and Rhoads, 2013; Key and Sneeringer, 2014).

Heat stress damages the barrier function of multiple epithelia and may increase the susceptibility to environmental toxicants (Zhang et al., 2020; Ma et al., 2022; Wu et al., 2023; Sun et al., 2024). For example, firefighters with elevated core temperatures have enhanced toxicant absorption, which increases the carcinogenic risk even with brief, high-level exposures (Bourbonnais et al., 2013; Jalilian et al., 2019; Mazumder et al., 2023). Heat stress-induced intestinal epithelial damage originates from enteric vasoconstriction and peripheral vasodilation, resulting in hypoxia, ATP depletion, and oxidative stress of the splanchnic tissues (Lambert, 2009; Mayorga et al., 2021). Additionally, hyperthermia disrupts the tight junctions and adherens junctions, increasing paracellular permeability and facilitating the translocation of toxicants into local and systemic circulation (Lian et al., 2020).

Although PFAS exposures have been associated with immunological and reproductive toxicity in humans and laboratory animal models, the effects of dietary PFAS exposures in animal agriculture are poorly understood. Consequently, this study investigated the tenet that PFOA exposure in post-pubertal pigs would alter immune, metabolic, and phenotypic parameters. Further, it was hypothesized that HS would exacerbate dietary PFOA’s adverse effects. Therefore, study objectives were to evaluate the impact of dietary PFOA, alone and in combination with HS, on growth performance, metabolism, organ weights, and biomarkers of hepatic health in reproductively synchronized post-pubertal pigs.

## Materials and Methods

### Experimental design

All procedures were approved by the Iowa State University Animal Care and Use Committee (IACUC #23-056). A recently published companion paper (Good et al., 2025) presents additional data from this study as part of a broader investigation. Forty-eight crossbred post-pubertal gilts (164.0 ± 11.6 kg body weight [BW]; PIC 1050 × PIC 337) were utilized in an experiment conducted at the Iowa State University Swine Nutrition Farm Research Facility (Ames, IA). Pigs were randomly assigned to 1 of 4 groups: 1) thermoneutral (TN) control (TN-CON; *n* = 12), 2) TN and PFOA (TN-PFOA; *n* = 12), 3) HS control (HS-CON; *n* = 12), or 4) HS and PFOA (HS-PFOA; *n* = 12). Pigs were allocated to 1 of 2 environmentally controlled rooms, each containing 24 individual crates (57 × 221 cm). Each crate had access to a stainless-steel feeder and a nipple waterer. Throughout the study, pigs were fed a corn and soy-based diet formulated to meet or exceed the requirements for essential amino acids, minerals, and vitamins (Supplementary Table S1; NRC, 2012). Feed and water were provided ad libitum during the entire experiment.

**Figure 1.**
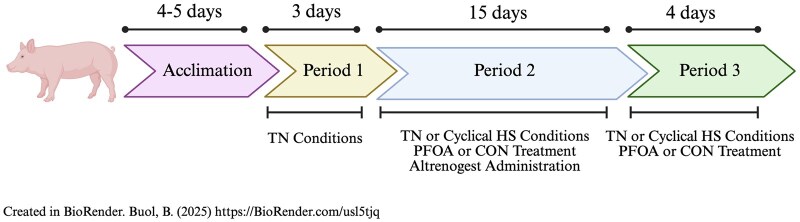
Schematic of the experimental design. Following acclimation (4 to 5 d), animals were assigned to Period 1 (3 d), Period 2 (15 d), and Period 3 (4 d) under thermoneutral (TN) or cyclical heat stress (HS) conditions with control (CON) or perfluorooctanoic acid (PFOA) treatment. Altrenogest was administered as indicated. Created with BioRender.

Prior to P1, pigs were moved into their respective environmental rooms and allowed to acclimate to their pens. The study consisted of three experimental periods (P): P1, P2, and P3 (Figure 1). During P1 (3 d), all pigs were housed in TN conditions (20.3 ± 0.1 °C; 44.5 ± 3.9% relative humidity [RH]) for baseline collection of production parameters and body temperature indices, and began receiving control cookie dough as a vehicle in preparation for PFOA dosing during P2 and P3. During P2 (15 d), TN-CON and TN-PFOA pigs remained in TN conditions (20.5 ± 0.3 °C; 49.9 ± 4.7% RH), while HS-CON and HS-PFOA pigs were exposed to a progressive cyclical HS with temperatures ranging from 26.6 to 32.2 °C (d 1 of P2: 25.7 ± 2.6 °C, 39.1 ± 3.2% RH from 0800 to 1800 h and 27.1 ± 0.1 °C, 32.7 ± 1.5% RH from 1800 to 0800 h; d 2 of P2: 29.3 ± 0.9 °C, 36.0 ± 1.9% RH from 0800 to 1800 and 27.0 ± 0.1 °C, 34.4 ± 0.8% RH from 1800 to 0800 h; d 3 of P2: 30.5 ± 0.9 °C, 36.0 ± 1.8% RH from 0800 to 1800 h and 28.2 ± 0.1 °C, 32.6 ± 1.4% RH from 1800 to 0800 h; and from d 4 of P2 to d 4 of P3: 31.9 ± 0.8 °C, 36.1 ± 2.2% RH from 0800 to 1800 h and 29.3 ± 0.1 °C, 33.5 ± 1.2% RH from 1800 to 0800 h). During the experiment, ambient temperature and relative humidity were monitored and recorded every 5 min by a data logger (Lascar EL-USB-2-LCD, Erie, PA), which was positioned down the center of the room (6 data loggers total), and data were then condensed by room into hourly averages.

Cookie dough was the vehicle for administering PFOA (CAS # 335-67-1; Sigma-Aldrich, St Louis, MO). Treatments were orally administered once daily (0800 h) and consisted of 10 g of cookie dough with no PFOA (CON) or with 70 ng/kg BW PFOA homogenized within the cookie dough. The daily PFOA dose was selected based on the drinking water level (ie, 70 parts per trillion) previously established as a health advisory level in drinking water for humans by the U.S. Environmental Protection Agency. A standard daily PFOA dose was calculated based on the average BW (164 kg) of all 48 animals obtained at the beginning of P1; thus, each pig received 11.48 μg PFOA/d which is similar to the maximum concentration (11.0 μg/L) of PFOA detected in drinking water (Crone et al., 2019; Kaiser et al., 2021). During P2, all pigs received an oral drench of Altrenogest (6.8 mL/d; Matrix, Merck Animal Health, NJ) for 15 d to synchronize estrus. During P3 (4 d), all pigs stopped receiving Altrenogest to facilitate estrus, but all other experimental (CON and PFOA) and environmental (TN and HS) treatments remained the same as P2.

Basal tissue PFOA quantification have been previously reported (Good et al., 2025). The basal feed and water PFOA concentrations were below the detectable limit (Good et al., 2025); thus, the PFOA administered via the cookie dough was the only demonstrable source of PFOA.

### Body temperature measurements

During P1, P2, and P3, body temperature indices (rectal temperature [T_R_], skin temperature [T_S_], and respiration rate [RR]) were obtained twice daily (0700 and 1800 h). Rectal temperature was measured using an electronic thermometer (SureTemp Plus 590, accuracy: ± 0.1 °C; WelchAllyn, Skaneateles Falls, NY). Skin temperature was measured at the rump using an infrared thermometer (IRT207: The Heat Seeker 8:1 Mid-Range Infrared Thermometer, accuracy: ± 2 °C; General Tools, New York, NY) ∼30.5 cm from the skin. Respiration rate was determined by counting flank movements for 15 s and later transformed to breaths per minute (bpm).

### Production parameters

Acclimation, P1, P2, and P3 feed intake (FI) was measured daily at 0700 h as feed disappearance. Body weights were obtained at the beginning of acclimation, P1, P2, and P3, and immediately before euthanasia. Average daily gain (ADG) and feed efficiency (gain: feed; G: F) were calculated by period.

### Blood sampling and analysis

Blood samples were obtained by jugular venipuncture (plasma: 10 mL K_2_EDTA vacutainers, Franklin Lakes, NJ; serum: 10 mL plastic serum vacutainers, Franklin Lakes, NJ; complete blood count [CBC]: 3 mL K_2_EDTA vacutainers, Monroe, NC) at the end of P1 and P2, and immediately before euthanasia. For CBC analysis, whole blood in K_2_EDTA tubes were submitted to the Iowa State University’s Department of Veterinary Pathology (Ames, IA) for automated-differential analysis using a flow cytometry-based hematology analyzer (ADVIA 2120i; Siemens, Munich, Germany). Plasma and serum samples were harvested and processed by centrifugation at 1,500 × *g* for 15 min at 4 °C, aliquoted, and stored at −80°C until further analysis. Plasma insulin, non-esterified fatty acids (NEFA), and glucose were determined using commercially available kits (insulin, Mercodia AB, Uppsala, Sweden; NEFA, FUJIFILM Wako Chemicals USA, Richmond, VA; glucose, FUJIFILM Wako Chemicals USA, Richmond, VA). The intra- and inter-assay coefficients of variation for insulin, NEFA, and glucose were 5.4 and 4.8%, 4.7 and 11.2%, and 2.7 and 2.6%, respectively. Biomarkers of hepatic function and health were analyzed with the VetScan VS2 Chemistry Analyzer using the Mammalian Liver Profile reagent rotor (Zoetis, Parsippany-Troy Hills, NJ).

### Tissue collection and liver triglyceride content analysis

Pigs were euthanized at the end of P3 via captive bolt technique, followed by exsanguination. The liver, lungs, left and right kidneys were immediately harvested, and organ weights were recorded. The carcasses were incinerated following tissue collections. Samples of liver tissue near the portal vein were snap-frozen in liquid nitrogen and stored at −80°C until analysis. Hepatic triglyceride (TG) content was measured as previously described (Horst et al., 2020). Briefly, approximately 25 mg of liver was homogenized with 750 μL of chilled PBS. The homogenate was centrifuged at 8,000 × *g* for 2 min at 4 °C and 300 µL of the supernatant was removed for free glycerol analysis and total protein content. An additional 400 μL of supernatant was removed and incubated with 100 μL of lipase (porcine pancreatic lipase, MP Biomedicals, Solon, OH) at 37 °C for 16 h. Free and total glycerol were determined via an enzymatic glycerol phosphate oxidase method (#F6426; Sigma-Aldrich, St Louis, MO). Total protein was determined via the bicinchoninic acid (BCA; Thermo Fisher Scientific, Waltham, MA). Free glycerol (before lipase digestion) was subtracted from total glycerol (after lipase digestion) to determine TG content, and this was expressed as a percentage of wet weight and as mg/g of protein of the original tissue sample. The intra- and inter-assay coefficients of variation for free glycerol and total glycerol were 5.3 and 0.5%, and 4.6 and 0.4%, respectively.

### Statistical analysis

The effects of dietary treatment (CON and PFOA) and environment (TN and HS) were analyzed as a two-by-two factorial using the MIXED procedure of SAS version 9.4 (SAS Inst. Inc., Cary, NC). For variables with multiple measurements over time (ie, FI, T_R_, T_S_, and RR), a repeated-measure analysis with an autoregressive covariance structure, and day as the repeated effect was used to determine the effects of treatment, environment, day, and their interactions. For variables recorded once per period, data were analyzed using a diagonal covariance structure with fixed effects of treatment and environment. Each parameter P1 value (when available) served as a covariate. Results are reported as least squares means and were considered significant with *P* ≤ 0.05 and a tendency when 0.05 < *P* ≤ 0.10.

## Results

### Serum PFOA

To confirm successful PFOA administration, circulating plasma PFOA concentrations were measured throughout the study. Across all timepoints, CON animals had PFOA concentrations below the limit of quantification. At the end of P2, HS-PFOA had markedly higher PFOA concentrations than TN-PFOA (1,780 vs. 1,180 ng/l, respectively; *P* < 0.01; Good et al., 2025). Thus, our hypothesis that HS increases the susceptibility to PFOA was supported. However, prior to euthanasia, plasma PFOA levels were similar in both treated groups (1,830 ng/l; Good et al., 2025). Tissue PFOA concentrations followed a similar pattern to circulation and have been recently reported (Good et al., 2025).

### Body temperature indices

**Figure 2.**
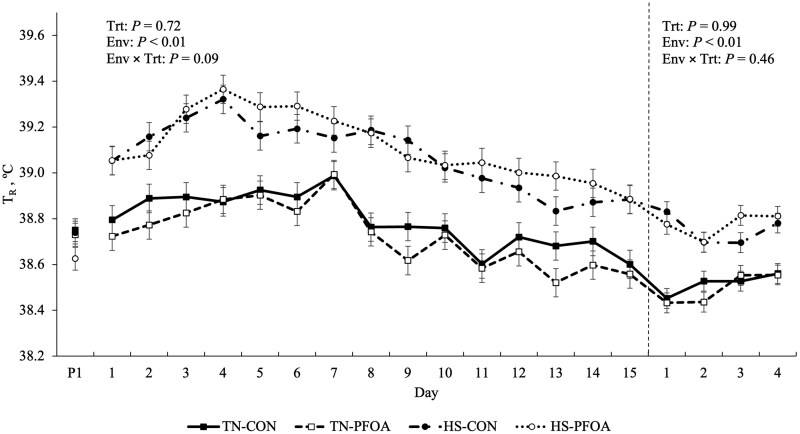
Effects of perfluorooctanoic acid (PFOA) on rectal temperature (T_R_) during periods 2 and 3. P1 represents the average of rectal temperature obtained during the 3 d of period 1. Treatments: TN-CON = thermoneutral (TN) control, TN-PFOA = TN and fed the PFOA treatment, HS-CON = heat stress (HS) control, HS-PFOA = HS and fed the PFOA treatment. The dashed line divides P2 from P3. Data are represented as least squares means ± standard error of the mean and considered significant if *P* ≤ 0.05 and a tendency if 0.05 < *P* ≤ 0.10.

During P2, pigs exposed to HS had an overall increase in T_R_, T_S_, and RR compared to their TN counterparts (0.34 °C, 5.67 °C, and 22 bpm; *P* < 0.01; Figure 2, Table 1). No T_R_, T_S_, or RR differences were detected between CON and PFOA animals during P2 (*P* > 0.11). There was a tendency for a treatment by environment interaction in T_R_ during P2 (*P* = 0.09), as PFOA mildly reduced T_R_ in TN conditions, but did not influence T_R_ during HS (Figure 2). Similarly, a treatment by environment interaction occurred with T_S_ as PFOA decreased T_S_ in TN, but did not affect T_S_ in HS pigs (*P* = 0.01; Table 1). During P3, HS increased T_R_, T_S_, and RR (0.26 °C, 6.96 °C, and 22 bpm; *P* < 0.01; Figure 2, Table 1) compared to TN and these variables were not influenced by PFOA (*P* > 0.34).

**Table 1.** Effects of perfluorooctanoic acid on body temperature indices and growth performance in thermoneutral and heat-stressed pigs

	Thermoneutral		Heat Stress			*P*-value		
Parameter	CON	PFOA	CON	PFOA	SEM	Trt^1^	Env^2^	Trt × Env
**Period 2**								
** T_S_ ^3^, °C**	36.5^b^	36.1^c^	41.9^a^	42.1^a^	0.1	0.23	<0.01	<0.01
** RR^4^, bpm**	42	41	65	61	1	0.11	<0.01	0.17
** G: F^5^**	0.34	0.30	0.31	0.30	0.02	0.39	0.44	0.55
** FBW^6^, kg**	189	186	181	181	1	0.22	<0.01	0.42
**Period 3**								
** T_S_ ^3^, °C**	36.1	35.7	42.8	42.9	0.2	0.34	<0.01	0.20
** RR^4^, bpm**	35	35	59	54	2	0.34	<0.01	0.40
** G: F^5^**	0.03	-0.11	−0.03	−0.29	0.08	0.02	0.17	0.43
** FBW^6^, kg**	189	185	181	178	1	0.01	<0.01	0.59

1 Treatment (CON: control or PFOA: perfluorooctanoic acid [70 ng/kg BW per day]).

2 Environment (thermoneutral or heat stress).

3 Skin temperature averaged by day.

4 Respiration rate averaged by day.

5 Gain to feed intake ratio.

6 Final body weight.

a–c Means with different superscripts denote an overall treatment difference (*P* ≤ 0.05).

### Production parameters

**Figure 3.**
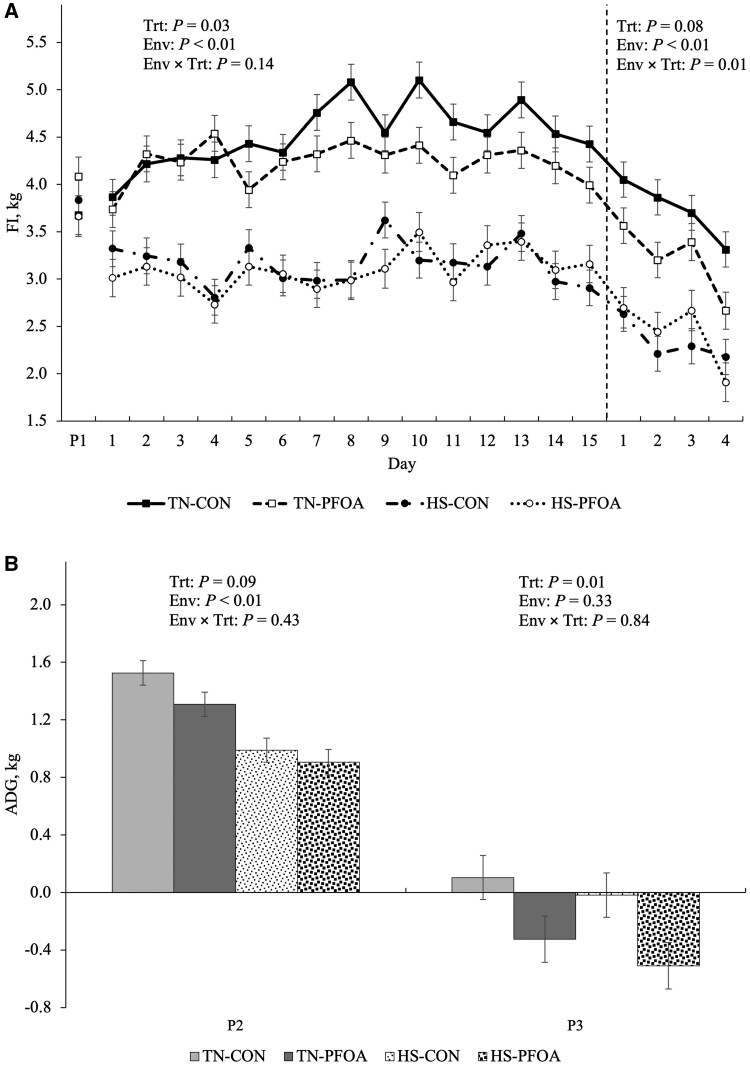
Effects of perfluorooctanoic acid (PFOA) exposure on (A) feed intake and (B) average daily gain during P2 and P3. Treatments: TN-CON = thermoneutral (TN) control, TN-PFOA = TN and fed the PFOA treatment, HS-CON = heat stress (HS) control, HS-PFOA = HS and fed the PFOA treatment. Data are represented as least squares means ± standard error of the mean and considered significant if *P* ≤ 0.05 and a tendency if 0.05 < *P* ≤ 0.10.

During P2, HS decreased FI (28.5%; *P* < 0.01; Figure 3A) relative to TN pigs. Overall, feeding PFOA also reduced FI (4.7%; *P* = 0.03; Figure 3A) during P2. ADG was reduced in HS compared to TN pigs (33.1%; *P* < 0.01), and PFOA tended to decrease ADG compared to CON pigs (11.9%; *P* = 0.09; Figure 3B). No treatment or environmental effects were observed in G: F during P2 (*P* > 0.39; Table 1). At the end of P2, BW was reduced in HS compared to TN pigs (6.5 kg; *P* < 0.01; Table 1).

In P3, there was a treatment by environment interaction on FI, where PFOA decreased FI in TN (0.52 kg; *P* = 0.01; Figure 3A), but did not affect FI in HS pigs. No environmental effects on ADG were observed in P3 (*P* = 0.33); however, PFOA pigs lost weight (0.46 kg; *P* = 0.01; Figure 3B) while ADG was nearly static in CON. During P3, feed efficiency was similar between environments (*P* > 0.17), but was decreased in PFOA-treated animals (0.20 units; *P* = 0.02; Table 1). During P3, final body weight (FBW) was decreased in HS compared to TN pigs (7.5 kg; *P* < 0.01; Table 1). Additionally, pigs fed PFOA had decreased FBW compared to CON pigs (3.5 kg; *P* = 0.01; Table 1).

### Circulating blood metabolism

During P2, no differences in blood glucose or insulin were detected across treatments or environments (*P* ≥ 0.36; Table 2). The insulin: FI increased during HS (39.2%; *P* = 0.01; Table 2), but was unaffected by PFOA. Circulating NEFA did not differ among treatments in P2 (*P* > 0.58), but HS tended to decrease NEFA (12.8%; *P* = 0.09; Table 2) compared to their TN counterparts. During P2, no treatment effects were observed in blood urea nitrogen (BUN; *P* > 0.48); however, HS reduced BUN relative to TN pigs (17.5%; *P* < 0.01; Table 2).

**Table 2.** Effects of perfluorooctanoic acid on blood metabolites in thermoneutral and heat-stressed pigs

	Thermoneutral		Heat Stress			*P*-value		
Parameter	CON	PFOA	CON	PFOA	SEM	Trt^1^	Env^2^	Trt × Env
**Period 2**								
** Glucose, mg/dL**	82	82	79	86	3	0.36	0.91	0.33
** Insulin, μg/L**	0.12	0.10	0.11	0.11	0.01	0.76	0.90	0.41
** Insulin: FI^3^**	0.026	0.025	0.034	0.037	0.004	0.77	0.01	0.51
** NEFA^4^, μE/L**	88	92	77	80	7	0.58	0.09	0.97
** BUN^5^, mg/dL**	11.54	12.30	9.89	9.79	0.47	0.48	<0.01	0.37
**Period 3**								
** Glucose, mg/dL**	108	118	96	100	8	0.37	0.07	0.74
** Insulin, μg/L**	0.12	0.13	0.08	0.08	0.02	0.57	0.02	0.72
** Insulin: FI^3^**	0.032	0.042	0.034	0.040	0.007	0.24	0.96	0.80
** NEFA^4^, μE/L**	160	167	105	119	24	0.66	0.04	0.87
** BUN^5^, mg/dL**	9.92	9.69	8.09	7.84	0.50	0.64	<0.01	0.98

1 Treatment (CON: control or PFOA: perfluorooctanoic acid [70 ng/kg BW per day]).

2 Environment (thermoneutral or heat stress).

3 Insulin to feed intake ratio.

4 Non-esterified fatty acids.

5 Blood urea nitrogen.

In P3, plasma glucose remained similar between CON and PFOA pigs (*P* > 0.37), but tended to be decreased in HS relative to their TN counterparts (13.3%; *P* = 0.07; Table 2). At the end of P3, circulating insulin did not differ between CON and PFOA pigs (*P* > 0.57), but was decreased in HS compared to their TN counterparts (36%; *P* = 0.02; Table 2). No treatment or environmental effects were observed for the insulin: FI during P3 (*P* > 0.24). Circulating NEFA did not differ between CON and PFOA pigs in P3 (*P* > 0.66), however, it was reduced by HS (31.5%; *P* = 0.04; Table 2). During P3, no treatment effects were observed in BUN (*P* > 0.64), but HS reduced BUN relative to TN (18.8%; *P* < 0.01; Table 2).

### Hepatic enzymes

During P2, circulating alkaline phosphatase (ALP) decreased in HS relative to TN pigs (18.4%; *P* = 0.01; Table 3), but it was unaffected by PFOA (*P >* 0.96). Similarly, no treatment effects were detected in alanine aminotransferase (ALT) during P2 (*P* > 0.92); however, it was reduced in HS pigs compared to TN counterparts (17%; *P* < 0.01; Table 3). Gamma-glutamyl transferase (GGT) was increased in HS compared to TN pigs during P2 (12.7%; *P* < 0.01; Table 3), but it was unaffected by PFOA (*P* > 0.48). No differences in circulating bile acids (BA), total bilirubin (TBIL), albumin (ALB), or cholesterol (CHOL) were observed due to treatment or environment during P2 (*P* ≥ 0.17; Table 3).

**Table 3.** Effects of perfluorooctanoic acid on biochemical parameters in thermoneutral and heat-stressed pigs

	Thermoneutral		Heat Stress			*P*-value		
Parameter	CON	PFOA	CON	PFOA	SEM	Trt^1^	Env^2^	Trt × Env
**Period 2**								
** ALP^3^, U/L**	91.4	92.6	76.0	74.2	5.9	0.96	0.01	0.80
** ALT^4^, U/L**	40.3	40.5	33.5	33.6	1.4	0.92	<0.01	0.94
** GGT^5^, U/L**	32.9	32.7	36.1	37.8	1.1	0.48	<0.01	0.39
** BA^6^, μmol/L**	3.62	1.53	3.21	3.36	0.98	0.33	0.47	0.27
** TBIL^7^, mg/dL**	0.31	0.30	0.32	0.30	0.01	0.17	0.65	0.65
** ALB^8^, g/dL**	5.39	5.45	5.37	5.41	0.06	0.42	0.59	0.88
** CHOL^9^, mg/dL**	92.8	94.3	89.0	92.5	2.5	0.34	0.28	0.70
**Period 3**								
** ALP^3^, U/L**	99.1	94.2	76.8	78.7	5.7	0.79	<0.01	0.55
** ALT^4^, U/L**	42.1	42.3	33.6	33.3	1.4	0.98	<0.01	0.83
** GGT^5^, U/L**	36.9	38.2	39.5	42.0	2.0	0.34	0.11	0.76
** BA^6^, μmol/L**	3.73	2.38	1.55	1.76	0.95	0.55	0.15	0.43
** TBIL^7^, mg/dL**	0.30	0.35	0.31	0.31	0.02	0.21	0.38	0.21
** ALB^8^, g/dL**	5.43	5.58	5.36	5.43	0.06	0.06	0.06	0.45
** CHOL^9^, mg/dL**	80.6	83.9	81.3	83.0	2.5	0.34	0.97	0.75

1 Treatment (CON: control or PFOA: perfluorooctanoic acid [70 ng/kg BW per day]).

2 Environment (thermoneutral or heat stress).

3 Alkaline phosphatase.

4 Alanine aminotransferase.

5 Gamma glutamyl transferase.

6 Bile acids.

7 Total bilirubin.

8 Albumin.

9 Cholesterol.

During P3, no differences in ALP levels were observed between CON and PFOA-fed pigs (*P* > 0.79), but overall, it was decreased during HS (19.6%; *P* < 0.01; Table 3). Similarly, circulating ALT was reduced in HS compared to TN pigs in P3 (20.7%; *P* < 0.01; Table 3); however, ALT was unaffected by PFOA (*P* > 0.98). Circulating ALB at the end of P3 tended to be increased by PFOA (2%; *P* = 0.06; Table 3) and tended to be decreased in HS (2%; *P* = 0.06; Table 3). No other treatment or environmental effects were detected in circulating GGT, BA, TBIL, or CHOL during P3 (*P* ≥ 0.11).

### Blood hematology

At the end of the P3, no differences were observed in circulating red blood cells (RBC) between CON and PFOA treatments (*P* > 0.29), however, RBC was decreased by HS (5.5%; *P* = 0.01; Table 4). Hemoglobin tended to be decreased by HS (3.6%; *P* = 0.10; Table 4) but was unaffected by PFOA (*P* > 0.56). Hematocrit was decreased by HS (2.3%; *P* = 0.02; Table 4) and was unaffected by PFOA. Neutrophils were decreased (16.3%; *P* = 0.03), and eosinophils were increased (41.1%; *P* = 0.03; Table 4) by HS, but neither leukocyte was affected by PFOA. Circulating white blood cells (WBC), platelets, lymphocytes, monocytes, and basophils were not affected by treatment or environment (*P* ≥ 0.36).

**Table 4.** Effects of perfluorooctanoic acid on complete blood counts in thermoneutral and heat-stressed pigs at euthanasia

	Thermoneutral		Heat Stress			*P*-value		
Parameter	CON	PFOA	CON	PFOA	SEM	Trt^1^	Env^2^	Trt × Env
**RBC^3^, ×10^6^/μL**	8.37	8.40	7.75	8.10	0.17	0.29	0.01	0.37
**Hemoglobin, g/dL**	15.64	16.00	15.24	15.27	0.33	0.56	0.10	0.63
**Hematocrit, %**	49.53	50.22	47.13	48.07	0.97	0.41	0.02	0.90
**Platelets, ×10^3^/μL**	251	228	229	249	35	0.96	0.99	0.56
**WBC^4^, ×10^3^/μL**	17.64	18.31	17.24	17.58	1.09	0.65	0.61	0.88
**Neutrophils, ×10^3^/μL**	5.09	4.77	3.81	4.44	0.36	0.68	0.03	0.20
**Lymphocytes, ×10^3^/μL**	11.08	12.01	11.70	11.39	0.85	0.71	0.99	0.47
**Monocytes, ×10^3^/μL**	0.71	0.77	0.80	0.71	0.08	0.87	0.83	0.36
**Eosinophils, ×10^3^/μL**	0.50	0.53	0.69	0.76	0.09	0.56	0.03	0.86
**Basophils, ×10^3^/μL**	0.09	0.11	0.09	0.09	0.02	0.43	0.55	0.51

1 Treatment (CON: control or PFOA: perfluorooctanoic acid [70 ng/kg BW per day]).

2 Environment (thermoneutral or heat stress).

3 Red blood cells.

4 White blood cells.

### Organ weights and triglyceride composition

Regardless of PFOA exposure, both absolute and relative liver weights were or tended to be decreased in HS animals compared to TN counterparts (9.13%; *P* < 0.01; and 4.9%; *P* = 0.08; respectively; Table 5). Lung weights were reduced in PFOA-fed animals relative to CON (11.9%; *P* = 0.02), and lung weights were reduced in HS pigs compared to their TN counterparts (12.2%; *P* = 0.02; Table 5). Lung weight as a percentage of BW tended to be decreased in PFOA-fed pigs (*P* = 0.09) and reduced in HS compared to their TN counterparts (*P* = 0.09; Table 5). Regardless of PFOA treatment, kidney absolute and relative weight decreased in HS animals (11.9%; *P* < 0.01; 8.5% decrease; *P* = 0.01; respectively; Table 5). Liver TG content, either as a percentage of wet weight or relative to protein content, was unaffected by HS and PFOA (*P* ≥ 0.15). A post-hoc analysis revealed that liver TG content as a percent of wet weight tended to be increased in TN-PFOA relative to TN-CON (*P* = 0.07).

**Table 5.** Effects of perfluorooctanoic acid on organ weights in thermoneutral and heat-stressed pigs

	Thermoneutral		Heat Stress			*P*-value		
Parameter	CON	PFOA	CON	PFOA	SEM	Trt^1^	Env^2^	Trt × Env
**Liver**								
** Weight, g**	2,121	2,118	1,929	1,923	61	0.95	<0.01	0.98
** % of BW**	1.12	1.14	1.07	1.08	0.03	0.57	0.08	0.94
**Lung**								
** Weight, g**	1,239	1,120	1,117	955	58	0.02	0.02	0.71
** % of BW**	0.66	0.62	0.62	0.54	0.03	0.09	0.09	0.55
**Kidneys**								
** Weight, g**	492	494	435	434	14	0.99	<0.01	0.91
** % of BW**	0.261	0.268	0.241	0.243	0.008	0.57	0.01	0.77
**Liver**								
** TG^3^, % wet weight**	0.58	0.85	0.56	0.59	0.10	0.15	0.18	0.26
** TG, mg/g of protein**	40.9	53.2	40.8	40.8	6.6	0.36	0.35	0.36

1 Treatment (CON: control or PFOA: perfluorooctanoic acid [70 ng/kg BW per day]).

2 Environment (thermoneutral or heat stress).

3 Triglycerides.

## Discussion

Since the 1940s, per- and polyfluoroalkyl substances have been widely used in industrial and consumer products, and their chemical stability contributes to their environmental persistence. Elevated circulating PFAS have been correlated with adverse health effects, such as liver damage, thyroid dysfunction, cancer, obesity, reduced fertility, and endocrine disruption (Peritore et al., 2023; Li et al., 2025). In human blood samples, PFOA levels range from 3 to 5 ng/ml and have generally declined over time due to phase out efforts (Schwartz, 2000; Alves et al., 2014). In contaminated drinking water areas, however, blood concentrations can reach as high as 22,000 ng/ml (Olsen et al., 2009; Fu et al., 2016). In wild and domestic swine, up to 1505 µg/kg and 335 µg/kg PFOA in liver, respectively, have been documented (Draghi et al., 2025). Thus, this study utilized the former health advisory drinking water levels established by the US EPA as an anchor dose (70 ppt PFOA) to assess effects in TN animals and to determine any additional impact of HS thereon. Additionally, the pigs were synchronized in estrus to minimize ovarian and systemic endocrine variation.

Similarly, HS is an environmental issue that can threaten human health and jeopardize growth, productivity, and health in farm animals. Heat stress compromises epithelial barrier integrity, and specifically the intestinal barrier function (Lambert et al., 2002), allowing for luminal content translocation into portal and systemic circulation (Hall et al., 2001), which can trigger an energetically demanding inflammatory response that repartitions nutrients away from growth and production (Baumgard and Rhoads, 2013). Evidence demonstrates that HS increases the susceptibility to toxicants in multiple species (Leon, 2008; Nie et al., 2022; Verheyen et al., 2022), but this has not been thoroughly evaluated with PFOA exposure in pigs.

This study implemented a chronic, cyclical HS model to simulate a diurnal heat pattern, which markedly elevated T_R_, T_S_, and RR; changes consistent with previous reports (Abuajamieh et al., 2018; Mayorga et al., 2018). Interestingly, an interaction between treatment and environment was observed for T_R_ and T_S_, wherein PFOA mildly decreased these parameters only in TN conditions. While statistically significant, the biological relevance of this observation remains unclear, but is likely due to PFOA-induced reductions in FI and ADG (discussed below) decreasing whole body thermogenesis.

As expected, HS caused hypophagia, and this is a highly conserved species response to a thermal load (Baumgard and Rhodes, 2013). Reduced FI is presumably a strategy to minimize endogenous heat production as the heat increment of feeding generates a large amount of thermal energy (Mayorga et al., 2019). Interestingly, feeding PFOA also reduced FI, which was most noticeable in the TN-housed pigs. Although not extensively evaluated, reduced FI described herein corroborates rodent data where feeding PFAS also decreased FI (Loveless et al., 2006; Reckziegel et al., 2024). The hypophagic response has been proposed to be mediated by PFOA-induced increased uncoupling protein 1 (UCP1; Shabalina et al., 2015). However, theoretically increased UCP1 should increase T_R_, but while PFOA did not affect temperature indices during HS, it actually decreased T_R_ and T_S_ during TN conditions in P2. Thus, why PFOA decreased FI in this experiment is ill-defined.

During the preovulatory phase (P3), pigs in both environments had a progressive decrease in appetite, and although the magnitude is surprising, this has been observed in pigs (Rogan and Black, 2023) and women (Lyons et al., 1989; Buffenstein et al., 1995). Further, the hypophagic effects of PFOA continued during P3 in the TN pigs, but not in the HS pigs. Why PFOA negatively affects FI in TN but not HS is unclear, but this phenotypic response has clear deleterious implications for commercial pig production.

Heat-stressed pigs (P2) grew substantially slower than the TN controls, and this too was expected as it is a highly conserved response among mammals (Mayorga et al., 2018; Goo et al., 2019; Serviento et al., 2020; Wang et al., 2020). Overall, dietary PFOA decreased ADG in P2, but this was most pronounced in the TN pigs (Figure 3B), and the pattern mirrors the FI differences (Figure 3A). It is difficult to determine if decreased FI caused suboptimal ADG or vice versa, as energy expenditure (ie, growth) drives FI (Conrad et al., 1964). Similar to the large decrease in FI during P3, growth essentially stopped during the follicular stage of the cycle, and in fact, PFOA-fed pigs actually lost BW during this time frame (Figure 3B). The effects of PFOA on changes in BW are inconsistent amongst species (Du et al., 2019; Attema et al., 2022; Su et al., 2022), but the negative effects of PFOA on growth described herein would have enormous implications for the commercial swine industry.

Heat stress decreased both absolute and relative lung weights, and to our knowledge, this is the first study to report this effect. Whether this is caused by a reduction in pulmonary tissue mass or fluid loss (dehydration) remains unclear. Interestingly, PFOA did and tended to reduce both absolute and relative gross lung weight. Most studies evaluating PFOA focus on lung function and toxicity (Sorli et al., 2020; Wang et al., 2024; Zhou et al., 2025), and some have reported negative effects (Chen et al., 2012; Ahmad et al., 2021) while others did not (Heinle et al., 2019). It is unclear if the smaller lung size reported herein is accompanied by impaired function, but in theory, suboptimal lung size may limit heat dissipation, as panting is a key thermoregulatory mechanism in pigs (albeit T_R_ and T_S_ were unaffected by PFOA during HS).

While PFOA did not affect kidney weight, similar to lungs, it appears that gross kidney weight is infrequently evaluated (or at least reported) during toxicity experiments. Kidney weight was almost 60 g less in HS pigs, and this pattern was consistent with relative kidney weight. Decreased kidney size described herein appears to be underreported, though HS-related effects on renal function have been documented (Chapman et al., 2021a,b). This response is slightly surprising, as water consumption increases during HS and this presumably increases glomerular filtration rate, which is sometimes associated with renal volume (Ghaith et al., 2021; Guo and Zhao, 2023).

Aspects of hepatic physiology were key objectives of this experiment. Similar to previous research (Johnson et al., 2015; Ahmed et al., 2021), HS decreased and tended to reduce the absolute and relative liver size. Reduced liver size is comparable to the reduction in lung and kidney weight, and this presumably is a strategy to reduce endogenous metabolic heat production as the three organs disproportionately produce a lot of heat (especially on a per-weight basis). The change in HS-induced liver size was unaccompanied by altered fat content. Orally delivered PFOA had no impact on liver size, and utilizing the entire factorial statistical design, it did not have a detectable effect on hepatic fat content. However, a post hoc analysis just on the TN treatments indicated that PFOA tended to increase liver lipid concentration on a wet weight basis, and this is noteworthy as similar histopathological effects have been previously reported (Marques et al., 2021; González-Alvarez and Keating, 2023; Stoffels et al., 2023). Reasons why PFOA increases liver lipid content appear to be multifaceted (Wang et al., 2013; Gou et al., 2024), and this is relevant to animal agriculture as the liver is not only an economically valued organ but is also the master regulator of nutrient partitioning.

Independent of nutrient intake, HS markedly altered some aspects of post-absorptive metabolism. During P2, circulating glucose was similar among environmental conditions, which aligns with our previous work (Pearce et al., 2015; Mayorga et al., 2021). Although HS-exposed pigs were in a catabolic state (ie, decreased FI and a reduced BW), they had a biologically paradoxical increase in insulin: FI. Insulin is an acute and potent anabolic hormone and it has a role in activating and upregulating heat shock proteins. While these findings corroborate others (Wheelock et al., 2010; Abuajamieh et al., 2018; Mayorga et al., 2021), the mechanisms underlying increased insulin dynamics during HS are not well-defined. Interestingly, lipopolysaccharide (LPS) administration acutely increases circulating insulin in animal models (Waldron et al., 2006; Kvidera et al., 2017); therefore, HS reduced intestinal barrier function and the subsequent inflammatory response triggered by LPS translocation may stimulate insulin secretions as a means of supporting a robust immune response (Kvidera et al., 2017).

Heat stress markedly modifies lipid metabolism as hyperthermic animals typically fail to mobilize adipose tissue, and this is especially apparent when compared to TN animals on a similar plane of nutrition. Herein, HS pigs had reduced circulating NEFA, which is likely explained by insulin as it is a potent antilipolytic ligand (Baumgard and Rhodes, 2013). Additionally, HS pigs had decreased BUN, and while this corroborates previous reports (Vásquez et al., 2022), it contradicts others that observed no effects of HS on BUN in pigs (Mayorga et al., 2021), or increased BUN levels in heat-stressed heifers and cows (Ronchi et al., 1999; Shwartz et al., 2009). Reasons explaining the inconsistencies are not clear, but the decreased BUN described herein would agree with the increased insulin: FI, as insulin is also a potent inhibitor of skeletal muscle mobilization (Baumgard and Rhodes, 2013). Although some PFAS are positively correlated with increased BUN in humans and in dogs (You et al., 2022; Hall et al., 2023; Su et al., 2025), there was no impact of PFOA exposure on BUN, nor any interaction between PFOA and HS.

Reductions in RBC, hemoglobin, and hematocrit may correlate with an increased hydration status (although not technically measured) during HS. Similar declines in these parameters have been reported in pigs (Mendoza et al., 2017) and dairy calves with pre- and post-natal HS exposure (Marrero et al., 2021). Furthermore, HS decreased neutrophils and elevated eosinophils, and while this disagrees with our previous work (Mayorga et al., 2021; 2024), it is difficult to utilize changes in circulating leukocytes as an indication of immune activation as their transient concentration represents a balance between entry and exit from the circulating pool (Mayorga et al., 2024). As for PFOA, human epidemiological studies report an association between PFAS exposure and hematological indices (Lopez-Espinosa et al., 2021; Lin et al., 2024). Indeed, PFAS are documented immunotoxicants (Hu et al., 2012; Phelps et al., 2023), yet no PFOA effects on CBC metrics were observed in the present study, and based on the available literature, no clear diagnostic values have been established. Consequently, relying on standard hematology panels in production livestock may underestimate the systemic burden of toxicants like PFOA.

To the best of our knowledge, this is the first study to investigate the interactive effects of HS and PFOA exposure in a livestock and large animal model. While additional research is warranted to validate our discoveries, the pragmatic implications of PFAS exposure may represent an enormous economic concern to animal agriculture. However, commonly used diagnostic indicators such as body temperature indices, blood metabolites, liver enzymes, and CBC parameters were largely uninformative in detecting PFAS exposure. Nonetheless, PFOA-exposed pigs had reduced FI and decreased growth. Though observed over a relatively short exposure period, these effects may be magnified in longer-term production scenarios. Future work is needed to confirm these outcomes and to elucidate the underlying physiological mechanisms driving the effects of HS and environmental toxicant exposure.

## Conclusion

We investigated the effects of HS and PFOA exposure in a post-pubertal pig model and discovered that even short-term PFOA exposure impairs production performance without triggering overt signs of pathology. Despite little to no effects on circulating biomarkers, these data support a growing body of evidence indicating that PFAS may compromise animal health and productivity. Heat stress reduced animal growth and productivity and all these effects were expected. As hypothesized, HS did appear to increase PFOA absorption. However, the negative effects of PFOA and HS on appetite, growth, and organ weights were seemingly independent of each other. Regardless, the burden of PFAS exposure and increased susceptibility to environmental stressors during HS may pose a substantial challenge for animal production systems.

## Supplementary Material

skaf348_Supplementary_Data

## Abbreviations:

ADG: average daily gain
ALB: albumin
ALP: alkaline phosphatase
ALT: alanine aminotransferase
BA: bile acids
BPM: breaths per minute
BUN: blood urea nitrogen
BW: body weight
CBC: complete blood count
CHOL: cholesterol
CON: control
EPA: Environmental Protection Agency
FBW: final body weight
FI: feed intake
G: F: gain to feed ratio
GGT: gamma-glutamyl transferase
HS: heat stress
NEFA: non-esterified fatty acids
P: period
PFAS: per- and poly-fluorinated substances
PFOA: perfluorooctanoic acid
RBC: red blood cells
RH: relative humidity
RR: respiration rate
TBIL: total bilirubin
TG: triglyceride
TN: thermoneutral
T_R_: rectal temperature
T_S_: skin temperature
WBC: white blood cells
