# Supplementary material for: Effects of perfluorooctanoic acid exposure and heat stress on performance and liver health biomarkers in post-pubertal gilts
Source: J Anim Sci. 2025 Oct 10;103:skaf348. doi: 10.1093/jas/skaf348 (PMC12597034; doi:10.1093/jas/skaf348)
Supplement: skaf348_Supplementary_Data [file skaf348_supplementary_data.docx]

| **Supplementary Table S1.** Ingredient composition of basal diet (as-fed basis). | |
| --- | --- |
| Item | % |
| Ingredient Composition |  |
| Corn | 78.15 |
| Soybean Mean, CP 46% | 9.82 |
| Corn DDGS^1^ | 10.00 |
| Lysine HCl | 0.22 |
| Threonine | 0.01 |
| Calcium | 0.15 |
| Vitamin-mineral premix^2^ | 1.65 |
| ^1^Corn distillers dried grains with solubles. ^2^Vitamin-mineral premix provided the following per kg of complete diet: 968.35 IU/kg of vitamin A, 133.69 IU/kg of vitamin D3, 4.67 IU/kg of vitamin E, 3.61 mg of pyridoxine, 0.90 mg of riboflavin, 2.67 mg of D-Pantothenic acid, 5.01 mg of niacin, 91.62 mg of Fe (ferrous sulfate), 102.66 mg of Zn (zinc sulfate), 22.08 mg of Mn (manganese sulfate), 10.67 mg of Cu (copper sulfate), 0.24 mg of I (calcium iodate), and 0.22 mg of Se (sodium selenite). | |
